# Internet gaming disorder: the four needs of the addiction

**DOI:** 10.3389/fpsyt.2024.1341140

**Published:** 2024-02-19

**Authors:** Sebastien Porcher

**Affiliations:** Ph.D, Heppenheim, Germany

**Keywords:** internet gaming disorder, self-determination, need, competence, autonomy, belongingness, escapism, therapy

## Introduction

### Context

Internet gaming has long been regarded as an ordinary leisure activity amongst others, until the growing number of addiction cases (Internet Gaming Disorder, IGD) [up to 27.5% of all internet gamers (1)] raised the interest of the media, due to the severe consequences associated with its excessive usage: sleeping problems, reduced social interactions or deep depression (2). After many years, this mediatization has finally, but only recently, found some echo with the World Health Organization, which listed this phenomenon in the International Statistical Classification of Diseases and Related Health Problems (3). This did however not convince the American Psychiatric Association, which continues to maintain this pathology in their Diagnostic and Statistical Manual of Mental Disorders (DSM-5) under the category “Conditions for Further Study”. As a consequence of this partial recognition, research on IGD has for years been confined within the model of a pathological evolution of the Motivation Factors for a leisure activity, delaying accordingly the emergence of other (broader) models such as those integrating cognitive aspects. Models which should have gained greater attention.

### The motivation factors of the internet game play

Since the emergence of IGD, investigations on its causes have been carried out primarily by searching for the motivations for a leisure activity, as inspired by the pioneer work of Nick Yee (4); leading to the identification of recurring Motivation Factors (5), namely:

-The social dimension: social interactions sparked by the video gameplay.
-The competition: enjoyment from challenges with other people.
-The skill development: the pleasure of improving skills, usually coordination in video gaming context.
-The recreation: the pleasure of playing.
-The achievement: the enjoyment of making progress in the game, to some extent related to the reward loop mechanism.
-The means to cope: approach to reduce the intensity of negative emotions or tensions, and to forget daily difficulties.
-The immersion: immerging into a different world, mostly full of fantasy.
-The fantasy: actions set in an imaginary world.
-The escapism: withdrawal from reality.

### The cognitive models of the internet game play

This stable, i.e. consolidated list of Motivation Factors, result from years of research, but has in recent years been challenged by some researchers (5) considering simple factors as insufficient for fully explaining the changeover into a dependance of what primarily a hobby is. From this view have emerged several cognitively integrative models such as PACE, which have recently, in a work of (6), been rationalized under the umbrella of the three psychological needs Self-Determination model (SDM) (7), composed of:

-The need for Autonomy: feeling of ownership.
-The need for Competence^1^: feeling of adequate capability.
-The need for Relatedness, or more accurately, the need for “Belongingness”. Indeed, “Relatedness” is usually mainly used for describing the genetical/genealogical connection between individuals (e.g. siblings, parents…), and not the human emotional need to interact with others, i.e. to be connected to a group in a quest for acceptance, as expressed in “Belongingness”.

## Discussion

### The limits of the motivation factor model

As shown above, the achieved maturity of the Motivation Factor view of IGD has raised initiatives for including it in a larger cognition-based framework, but despite this apparent inclusion, the two approaches continue to be perceived as two independent ones. This can be partly explained by the apparent absence of need dimension within the factors, a feature necessary for the emergence of dependance syndromes: salience, withdrawal, mood modification (irritability, conflict trigger…) (8) or the neglect of the most basic human obligations, like feeding a newborn baby, as reported from Korea with the case of Kim Sar.

### The needs of the self-determination model

On the basis of this analysis, we undertook to attribute to each Need the relevant Motivation Factors, a task we completed quite easily with respect to Autonomy and Competence (“Recreation”, “Achievement”, “Skill development”), but regarding Belongingness, no Motivation Factor seemed to suit this latter Need. The solution came when we realized that the emergence of the “Belongingness” should be described not only as the result of the initiatives of an individual towards a group for gaining acceptance, but also as all actions of an individual to be at the center of the group attention^2^. This view would explain many basic behaviors such as the use of make-up, the tattooing & piercing, some provocative attitudes, the attractiveness for social media, or the aspiration for wealth & power (CEO, politicians) (9), and for IGD would allow to elaborate a rational behind the dependance; a player in quest for the attention of other players (“Social”) will invest in deleterious i.e. addictive hours of practice (“Skill development”, “Competition”) for reaching the game mastering level that will give them the attention (i.e. will reward them) they look for.

### An extended self-determination model to encompass the escapism need

Having overcome this first difficulty, the second one arose: some Motivation Factors (“Coping”, “Immersion”, “Fantasy”, and “Escapism”) found no SDM Need to be associated with. This absence of connection was even more surprising since all these factors cover the same concept, the Escapism *“the way of avoiding an unpleasant or boring life, especially by thinking, reading, etc.”* [Cambridge Dictionary], an association already made by N.Yee (4) for the Immersion, and by (10), for the Coping.

Based on these considerations and the reported preponderance of Escapism in the development of the IGD (11), we came to the hypothesis that “Escapism” is a Need on its own (“*A thing or a feeling that when deficient causes a clear adverse outcome, a dysfunction or death”, according to the Cambridge Dictionary*), an assumption we could not support with any reference, since the notion has never been studied from this angle. Nevertheless, we were able to find one indirect evidence of its importance through the work of (12) on the “mind-disengagement”.

In their experiment, the authors left subjects alone in a room with absolutely nothing to do, except the possibility of administering electric shocks to themselves. At the end of the experiment (15 min of idleness) 43% of the subjects had administered at least one electric shock to themselves, confirming the difficulty for humans to maintain a disengaged mind, i.e. the necessity for humans to escape from the ground reality.

### The extended self-determination model, a predictive model

With the identification of this last element, and its adjunction to the SDM, we obtain a model covering all Motivation Factors, i.e. potentially able of prediction.

Therefore, while looking at game genres, we can expect that a form integrating the four Needs would be the most addictive i.e. having the highest proportion of IGD sufferers. And this is indeed what we observed with the MMPORG [28% of IGD-suffering players (13)], a game genre featuring the immersion in a virtual world (Escapism), integrating an exploration mode (Autonomy of decision), and with a multiplayer mode (Need of Attention, i.e. Belongingness).

Similarly, it can therefore be expected that people in high need of Autonomy will develop a dependence on any activity offering this, such as internet video games. ADHD sufferers belong to this category of persons (up to 39% of the IGD population) (14) who are known for their difficulty in dealing with social codes and constraints (15).

IGD is also known for its strong association with depression, commonly 32% and up to 75% of the subjects (16). This prevalence would be regarded as the result of playing internet video games as a means to fulfill the need of Escapism, rendered particularly high by the depression and the boredom associated with it. The fulfillment of the Escapism Need would also explain the motivation for the Hikikomori for playing (17).

### The limited efficacy of therapies explained

In the light of these arguments in favor of the involvement of Needs-fulfillment (Competence, Autonomy, Belongingness, Escapism) in the development of the IGD, the limited efficacy of therapies (18, 19) could be explained by their deficiency vis-a-vis the necessity of a Need-fulfillment. Under these considerations, only therapies axing their approach on the activity substitution^3^ but with the same type and level of fulfillment or putting their focus on the development of even stronger Needs, such as autonomous food supply, would have a chance to significantly influence the course of a IGD development.

## Conclusion and outlook

As we have seen, both the development & reinforcement of IGD are driven by the quest for the fulfilment of Needs − Competence, Autonomy, Belongingness (in a sense of attention seeking), and Escapism – i.e. driven by strong instincts which incidentally weaken the potential for therapies (e.g. cognitive-behavioral therapy, bupropion medication…) or prevention measures (e.g. boredom through extremely easiness of play, deployment of permadeath feature…) to reduce the number of IGD suffering people. By presently addressing the central role played by these Needs, we hope to raise a new interest for the systematization of the usage of the Need-fulfilling activities (e.g. sport activities) in the treatment and prevention of this disorder.

## Author contributions

SP: Writing – original draft.

## Footnotes

1 Accordingly, poor skill (and the resulting boredom) probably constitutes the best protection against the internet video game addiction.

2 It can be speculated that the Need of Attention is also at play in the maintenance of the perennity of the human species, toddlers depending for their survival on their capacity to attract the attention of the adults they depend on, and similarly, for reproduction, the connection with a sexual partner also depending on the capacity by an individual to attract the attention of another.

3 Not easy, the natural laziness of humans (energy preservation) renders any activity “demanding” i.e. unattractive (if not disgusting), in particularly in comparison with the easy-going ones offered by the internet video games play.
